# A rigorous approach to facilitate and guarantee the correctness of the genetic testing management in human genome information systems

**DOI:** 10.1186/1471-2164-12-S4-S13

**Published:** 2011-12-22

**Authors:** Luciano V Araújo, Simon Malkowski, Kelly R Braghetto, Maria R Passos-Bueno, Mayana Zatz, Calton Pu, João E Ferreira

**Affiliations:** 1 EACH - School of Arts, Sciences and Humanities, University of São Paulo, Rua Arlindo Béttio, 1000, Ermelino Matarazzo, São Paulo, Brazil; 2 CERCS, Georgia Institute of Technology, 266 First Drive, Atlanta, GA 30332-0765, USA; 3 Institute of Mathematics and Statistics, Computer Science Department, University of São Paulo, Rua do Matão, 1010, 05508-900, São Paulo, SP, Brazil; 4 The Human Genome Research Center, University of São Paulo, Rua do Matão, Travessa 13,106, 05508-900, São Paulo, SP, Brazil

## Abstract

**Background:**

Recent medical and biological technology advances have stimulated the development of new testing systems that have been providing huge, varied amounts of molecular and clinical data. Growing data volumes pose significant challenges for information processing systems in research centers. Additionally, the routines of genomics laboratory are typically characterized by high parallelism in testing and constant procedure changes.

**Results:**

This paper describes a formal approach to address this challenge through the implementation of a genetic testing management system applied to human genome laboratory. We introduced the Human Genome Research Center Information System (CEGH) in Brazil, a system that is able to support constant changes in human genome testing and can provide patients updated results based on the most recent and validated genetic knowledge. Our approach uses a common repository for process planning to ensure reusability, specification, instantiation, monitoring, and execution of processes, which are defined using a relational database and rigorous control flow specifications based on process algebra (ACP). The main difference between our approach and related works is that we were able to join two important aspects: 1) process scalability achieved through relational database implementation, and 2) correctness of processes using process algebra. Furthermore, the software allows end users to define genetic testing without requiring any knowledge about business process notation or process algebra.

**Conclusions:**

This paper presents the CEGH information system that is a Laboratory Information Management System (LIMS) based on a formal framework to support genetic testing management for Mendelian disorder studies. We have proved the feasibility and showed usability benefits of a rigorous approach that is able to specify, validate, and perform genetic testing using easy end user interfaces.

## Background

The production of large molecular and clinical datasets in modern biological research centers has posed new major challenges for information processing systems. In a typical biological laboratory routine, many tests are run in parallel by a single equipment; for instance, a thermal cycler or DNA amplifier can perform several different PCR reactions simultaneously. This high parallelism demands accurate procedure, reagent, and result management. New tests are being continually developed and users have to be guided to perform the right task at the appropriate time. Incompatibilities between new and old processes and new data requirements make it difficult to integrate and analyze all the available information. Besides, the process of scientific knowledge discovery involves frequent process updates for the refinement of scientific hypotheses. Since new data are automatically generated and processed, manual approaches have become very expensive or even infeasible. As a result, a long-term solution to the scientific data integration issue requires formally validated and automated information processing tools.

An illustrative example is genome databases that are characterized by large amounts of data that are frequently updated from a variety of data sources. It is a highly time-consuming process to integrate “new” genome data especially because the processes that create keep changing and software tools must be continuously updated to reflect these changes. As a result, delayed software evolution may slow down scientific progress.

### Challenges in the CEGH environment

The Human Genome Research Center (CEGH) created at the University of São Paulo is the largest center in Latin America dedicated to the study of human Mendelian genetic disorders. Since its establishment about 40 years ago, more than 100,000 patients and their relatives have been referred to the center and have been examined by different research groups. CEGH’s main mission is to gain understanding of gene function with a focus on neuromuscular, craniofacial, and brain development through the study of genetic disorders.

The CEGH offers around 40 different genetic tests, which are performed by several technicians under the supervision of six researchers. All samples are recorded and sent to specialized technicians for analysis. It is crucial to have a flawless control flow for each sample during every step of analysis.

One of the tests performed is polymerase chain reaction (PCR) with primers and amplification, specific for the segments (i.e., exons or introns) of a particular gene to be tested. The results are obtained through the analysis of PCR products. Most tests involve sequencing of several exons of the gene, which usually consists of a three-step procedure: PCR, DNA purification, and sequencing reaction. Note that amplification and reagents usually vary per exon and gene to be tested. Another set of tests requires a screening step using DHPLC or a different strategy, and only exons with an altered pattern in the first step are sequenced.

Genetic disorders are very heterogeneous and their phenotypes can be encoded by several genetic mechanisms involving more than one gene. Genetic testing is required for accurate estimates of recurrence risks. A strategy for cost reduction of testing is to perform common genetic tests that are used for most cases of the disorder being investigated. Once a negative result is obtained, a second test is performed, which is used for the second most frequent cause of the disorder. At times, three or more different tests need to be performed.

Besides the heterogeneity of diseases, there are several gene mutations that can cause a single disease. For example, there have been so far described more than 1,500 mutations for cystic fibrosis, which is the most common autosomal recessive disorder in Caucasians. As previously mentioned, testing starts in regions most likely to have mutations; other segments of the gene are tested only if negative results are obtained.

**Figure 1:**
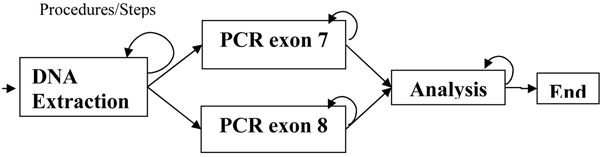
**Spinal muscular atrophy (SMA) test.** Illustration of how SMA genetic testing should be performed. The procedures of the test are represented in rectangles and the arrows indicate the order of execution of each step. This example is to illustrate the use of the proposed software.

Figure 1 shows patients, tests and their atomic steps, as well as the order of test performance. In other words, the steps shown in this figure are an illustration of test representation. This genetic test is used to identify mutations associated with spinal muscular atrophy (SMA) [1], a neuromuscular disease that causes progressive muscle degeneration. As most SMA patients carry a deletion of exons 7 and 8, the SMA test will probe the gene SMN1 looking for this type of mutation in exons 7 and 8. First, DNA is extracted from a patient sample. Then PCR of exon 7 and PCR of exon 8 can be performed. And because they are two independent procedures they can be performed simultaneously. At completion of both PCRs, the procedure analysis is released to execution, and it will analyze the previous results to produce a test result. Each procedure can be repeated as needed, as shown by a return arrow.

During laboratory routine, several tests are run at the same time by a single instrument requiring accurate procedure, reagent, and result management. Since new genetic tests are constantly being developed from old ones, a software system is required to handle various different versions of procedures and tests. Moreover, users have to be guided to perform the right task at the appropriate time.

### Related work

Previous research efforts in scientific data management have adopted several foundational concepts and tools to address the challenges of biological testing management. A popular approach is to include ontology evaluation into the design and specification process of biological system rules [2-4]. However, ontologies cannot include semantics of tests and effectively address dynamic changes in genome testing routines. Several successful efforts have been documented with automatic generation of scientific workflows through technical planning based on ontology descriptions [5-7]. As a downside, these approaches do not support the control of long transactions. An alternative approach is to use software architecture for knowledge discovery in biology [8], but its focus is solely on the data analysis of the process under study.

Although there are several tools for the management of workflows and business processes, some of them are focused on providing resources for process or work flow simulations. In other cases, processes can be managed using software tools without a formal validation. It becomes especially critical as process complexity increases. Some popular tools for the control and implementation of scientific workflows and business processes have been developed based on formal approaches such as colored Petri nets [9-12] and process algebra [13-15].

In fact, workflow approaches have been applied to develop the Laboratory Information Management Systems (LIMS) as an efficient method to handle tests performed in the laboratory.

The Protein Information Management System (PIMS) [16] and the WIST: Toolkit for rapid and customized LIMS development [17] are examples of programming language approach that offers an easy interface to define laboratory process routines based on specific patterns and manage workflow requirements. The PIMS is a specialized LIMS to manage protein testing methods with a customized notation to define workflow for protein analysis. The WIST provides a set of application programming interfaces and web application to support the LIMS development. The PIMS and WIST are a group of software approaches that uses specific workflow definition languages and workflow management systems to support laboratory management requirements. These approaches have been successfully used in several information systems offering a customized workflow solution.

Another important development of the LIMS approach is based on integration software tools to reduce the time, complexity and cost of software development. A representative example of this approach is the SIGla: an adaptive open source LIMS for multiple laboratories [18] that provides the integration between a generic information system and several available workflow engines using standard files. SIGla provides workflow features using the Enhydra Shark [19] open source editor that is a graphical tool to design workflows. Once the workflow is designed, SIGla creates a XPDL file [20] allowing workflow execution into a workflow engine, called Shark. In addition to the use workflow management tools, the Shark framework in SIGla has been expanded to support important features including sequential chaining (where the output of an activity can be used as input to another activity), and repetition procedures for exception handling. The SIGla authors presented an example where the integration of workflow tools was not a simple task. To make this integration possible, some cases require not only adaptation but also the development of new features.

Both LIMS approaches (i.e., programming language and workflow engine integration) provide scalability since there are many ways to integrate and adapt laboratory management systems. However, they do not support explicitly control-flow algebraic properties for workflow systems. The lack of algebraic properties is a disadvantage when there are involved complex workflows and mission-critical laboratory routines.

In this paper, we present an alternative LIMS approach that allows formal validation of workflow generated by the CEGH interface based on process algebra. This formal approach not only supports evaluation but also can be used for the execution of workflow applications.

## Implementation

The proposed approach was developed within the context of Process-Aware Information Systems (PAIS) that can be used to avoid hard-coding the processes management into tailor-made applications and thus support the shift from programming to assembling [21]. More specifically, our approach can provide scalability (through relational database) and correctness (through process algebra) of processes using an intuitive integration of (e.g., genomic) process control and classic information systems. We are able to share patient data, medical, laboratory and disease information, clinical annotations, and access control. Users can define genomic procedures using specific interfaces. Subsequently, our information system can automatically generate algebra expressions and use these expressions to control routine laboratory procedures.

A careful separation between process logic and application functionality is necessary to overcome these technological challenges. It is commonly known as workflow management and requires the identification of system functions as steps in a workflow process. Working under the premise of well-defined interfaces and modular implementation, this approach provides easy interfaces that can be modified without changing programming language codes. According to Fokkink definition [22], a workflow in the context of information systems can be defined as automation of a process in which information or tasks are passed from one entity to another according to specific process rules. A workflow management system defines, creates, and manages workflow execution with the use of software. Workflow engines are at the core of these systems and are able to interpret process definitions and interact with participants. As a result, workflow technology effectively integrates heterogeneous components and provides transparent control.

Although a wide variety of technologies may potentially be applicable all these systems are centered on the idea of effectively implementing process execution [23]. Any such system that controls a flow of steps or activities can be summarized under the umbrella of PAIS [24]. An emerging trend in PAIS is to embed processes in formal frameworks (e.g., process algebra or Petri Nets), which can be highly helpful during the entire process life cycle. Sound models enable the use of distinct sophisticated methods during verification, validation, diagnosis and reliable execution control. In this research project we applied our rigorous approach to event-based PAIS architecture in genetic testing in human genome information systems. A common repository of process planning ensures reusability, specification, instantiation, monitoring, and execution of processes. This repository is defined in relational database architecture. An application library helps implement a workflow engine as SQL extension for process instantiation, execution, control and monitoring using rigorous control flow specification based on the process algebra ACP.

### The navigation plan concept

Multiple application workflows are composed of actions that are performed through multiple applications, integrating data in heterogeneous information system environments. There are significant challenges due to potentially complex interferences among autonomously designed components. In order to address these challenges, we analyze each system transaction and divide it into three steps: 1) A client (i.e., human user or program) generates a request; 2) the request is validated through a series of activities — a legitimate request must meet a set of pre-specified requirements; 3) A validated request is passed to execution. This approach’s main assumption is that the three steps of order processing can be separated, implemented, integrated, and executed through well-defined interfaces, which support both integration of heterogeneous and autonomous information systems and workflows.

The Navigation Plan Concept [25] is defined through a clear compositional structure. A *single action* is a set of *atomic actions* formed using process algebraic operators such as sequential, alternative (+), and parallel that use the following notations: (.), (+), ( || ), respectively; a *checkpoint* is a set of atomic actions formed using a set of process rules (i.e., constraints and conditional rules); a *step* is either a single action or a checkpoint; a *process* is a set of steps formed using process algebraic operators (sequencing, alternative composition, recursion, and communication). A *navigation plan* is a set of all processes in an application required to achieve a system goal.

The main innovation of this concept is the ability to link semi-formal description using process algebra to a practical execution environment. On the formal side, process steps are mapped to process algebra for their composition. On the practical side, navigation plans are directly executed using RiverFish architecture which guarantees the properties predicted by the process algebra.

### The RiverFish architecture

The RiverFish architecture [26] is designed as a practical implementation of the Navigation Plan Concept. Its unique features include modularity, reusability of transactional components, and simplicity of data structures. The architecture can represent data and process steps according to ordering rules specified in the navigation plan. The three main components of RiverFish implemented are: unified control, instance execution, and data storage.

### Navigation plan definition language

The Navigation Plan Definition Language (NPDL) [27] uses process algebra [22] as a formal basis for process representation and can be considered as a rigorous process representation language for controlled execution. NPDL adopts concepts (i.e., actions and operators) from basic process algebra extended by ACP’s merge operator and recursive expressions. A process in NPDL is defined by a closed term, which is built from a set of atomic actions, operators and composed processes. For instance, the following NPDL commands specify a process called “compound process” that computes a conventional addition or multiplication of two numbers for an unrestricted number of times. The NPDL is a way to go from process algebra expression to a workflow system using a SQL extension. The following NPDL example is the input for a workflow engine called Navigation Plan Tool (NPTool) [28] that will control workflow execution.

In addition to these basic operators, NPDL also includes the interleaved parallel composition “|*”, the multi merge composition “&”, the discriminator composition “^”, the unlimited repetition “?*”, the number limited repetition “?*n*” (*n* is a positive integer), the function limited repetition “?*f*” (*f* is a function that returns a positive integer), and the conditional execution “%*r*” (*r* is a Boolean rule). These additional operators enable NPDL to adequately specify common control flow actions. It is important to note that the methods of analysis applied to process algebra expressions remain applicable for NPDL expressions. The NPDL operators “^,” “&,” “|*,” “?n,” and “?*” can equivalently be replaced by using the basic operators (i.e., “.,” “+,” and “||”) and recursion. The operators “%*r*” and “%!*r*” can be eliminated from the expressions, and “?*f*” can be replace by “?*” without compromising the analysis. The main goal of NPDL is to provide expressive and intuitive execution mechanisms for control flow patterns in corporate and research process environments. The required interpreter / execution engine is implemented in the Navigation Plan Tool. It emphasizes the sharing capabilities in cooperative environments and offers support through data structures implemented in Relational Database Management Systems (RDBMS).

### The navigation plan tool

The Navigation Plan Tool (NPTool) [28] controls process execution and is designed for integration in information systems. NPTool employs NPDL and a relational database to specify processes and control their instantiation and execution. The tool is implemented as RDBMS-independent SQL extension in J2SE 5.0 (i.e., JDBC enables standard SQL database access). It ensures an easy integration with traditional information systems, which generally have mechanisms that provide access to RDBMS. The storage of process data in the database adds scalability to the execution control provided by NPTool. Moreover, process definitions become easily reusable and the database maintained by NPTool can be viewed as a common process repository.

## Discussion

In this section we describe how the CEGH Information System uses process representation to manage genetic testing execution. This system has around 100 tables and 50 interfaces to manage patient and family data, medical, laboratory and disease information, clinical annotations, access control, tests, relationships between tests and diseases, testing order, their execution and analyses. It has been implemented in Ruby on Rails version 2.3 and uses a PostgreSQL database version 8.4 under a Linux environment. Details about the CEGH system are available at http://zen.genoma.ib.usp.br/. The system is implemented in Portuguese language but we have provided an English translation with a demo.

Briefly, tests are defined as a set of actions called procedures. The execution of these procedures will constitute the desired genetic analysis. In addition, a procedure describes the techniques applied in each test step, and it comprises all required information to run the entire procedure, for example, a list of reagents used.

**Figure 2:**
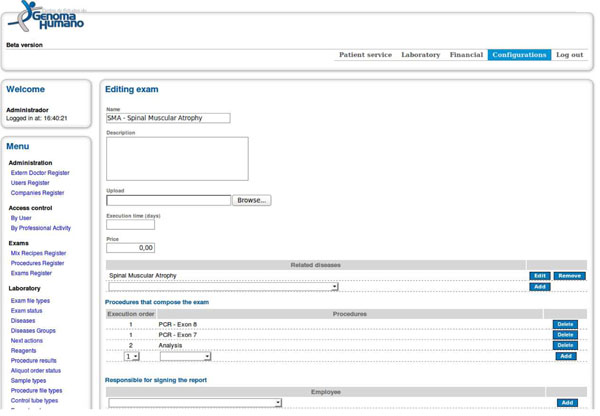
**User interface for the definition of genetic testing.** Interface for procedure definition and execution order of a genetic test. It is also automatically generates in a transparent way the process algebra expression corresponding to the test.

Going back to the example of test representation, Figure 1 illustrates a genetic test performed at the CEGH laboratory to identify mutations associated with SMA, and Figure 2 presents how the SMA test was defined. According to Figure 2, our software interface allows users to define how the genetic test must be executed. This is done without requiring process algebra knowledge. Instead of creating complex algebra expressions or using graphical tools to define the workflow, a user is only required to select the composition procedures of a genetic test and the execution order of each procedure. Based on this information, the software program is able to translate the user’s definitions into process algebra expressions. This interface is designed to meet the user’s needs and to be semantically close to the way he/she understands and recognizes the composition steps of a genetic test. It allows the use of a formal approach to manage processes without increasing software complexity for the end user.

At the top in Figure 2 we can see the fields for test description: name, description, file upload, test duration in days and cost. There is also a field to define the test process and test procedures are selected from a set of pre-defined procedures. As shown in Figure 2, the PCR exon 7 and PCR exon 8 procedures were selected to compose the SMA test. The execution order field indicates the priority of procedure execution. The procedures will be executed in an ascending order and procedures with the same priority will be run in parallel.

The test definition is saved and then the corresponding process algebra expression is created by NPDL engine. The expression generated by the software to represent the SMA test is presented below.

In this expression each procedure is represented as an atomic action and the execution order is represented using a process algebra operator. Procedures with the same execution order are mapped using the operator for parallel execution “||” and the link between procedures with different execution order number is mapped using the operator for sequential composition “.”. The conditional execution operator % assessed the successful procedure execution completion and released the execution of an associated action.

**Figure 3:**
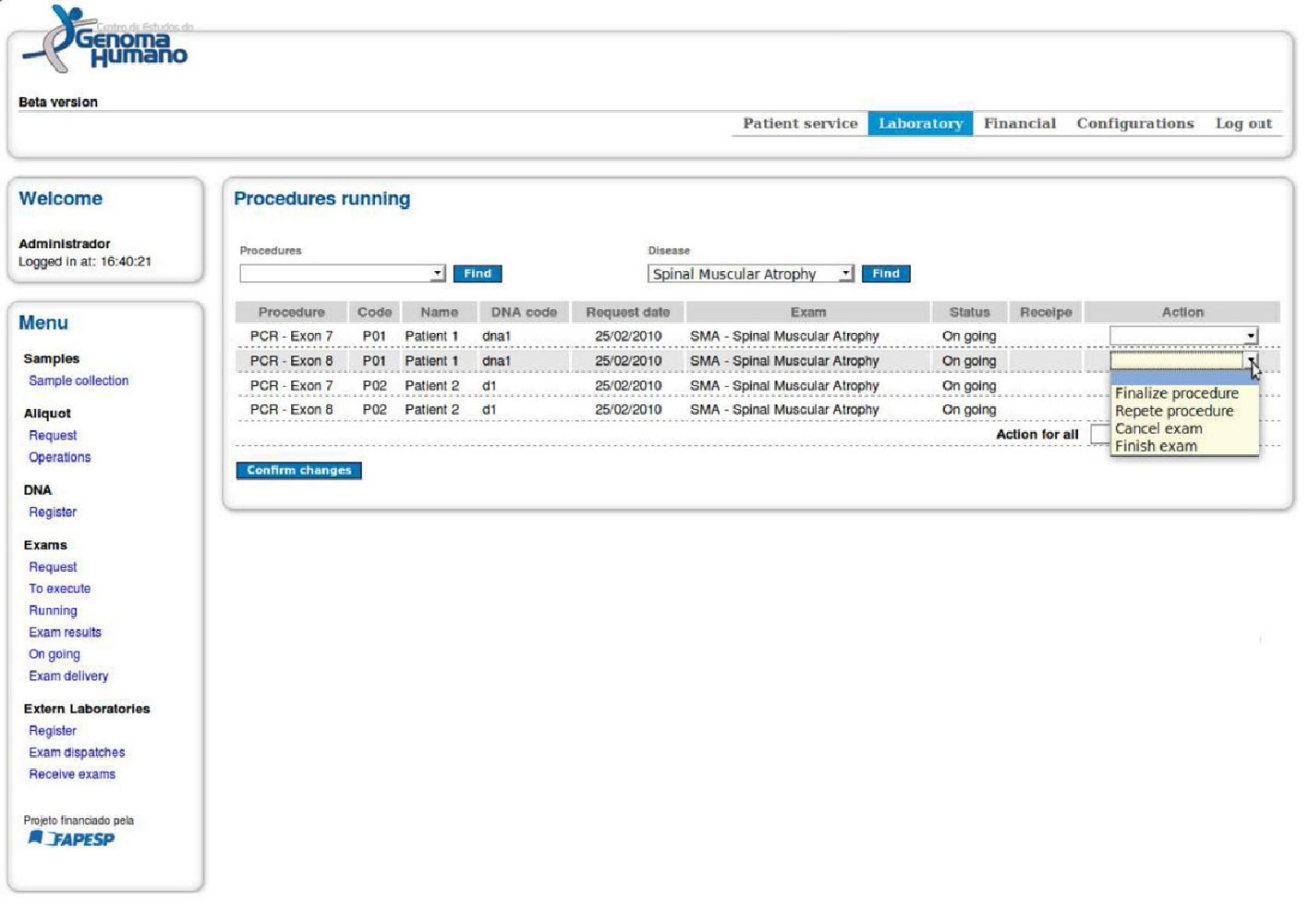
**User interface to control procedure execution and analysis.** This interface collects data on the execution of procedures of a test and defines the next procedure to be executed based on the interpretation of the process algebra expression.

The NPDL expression is used by the CEGH system to execute this SMA test. It indicates that PCR exon 7 and PCR exon 8 procedures can be run in parallel. The action associated with the procedures is a silent action GO (Go On) and it indicates that the expression verification must proceed. The atomic action END is released to execution at the completion of both procedures and it indicates that SMA test is complete. After the PCR exon 7 and PCR exon 8 procedures are completed and their results analyzed, the SMA test can be finished and the results stored. The next procedure to be performed is selected when using the interface shown in Figure 3 the user indicates the end of the execution of a procedure. At this point, the software will analyze the algebraic expression to indicate the next step to be executed. Thus, for each procedure, the user must inform the execution status as shown in Figure 3.

Figure 3 presents the interface to be used for the execution of procedures. First, users can monitor procedures and tests to which they have been granted permission.

The fields ‘procedure’ and ‘disease’ at the top of Figure 3 help the user to handle the parallel execution of tests, since independent procedures can be executed simultaneously in different equipment or laboratory areas. The CEGH interface offers two options to carry out the test. The first allows the execution of a group of similar procedures regardless of the test performed. The procedure to be carried out in this group of tests is selected in the field 'procedure’. For instance, if the user selects PCR in the field ‘procedure,’ the interface will present all types of PCR waiting for execution. This approach is also useful when the user have to perform many procedures in the same lab equipment. The second execution option allows following up a specific test. In this option, the user selects the desired test in the field ‘disease.’ Only the specific procedures of the selected test are displayed and are also released for execution through the analysis of the algebraic expression. A list of procedures is then presented. The procedures are described using the following fields: procedure name; patient number; patient name; DNA number; date of test request; test name; execution status; link to recipes for reagents; and action combo box. In the action combo box, a user can select the procedure status as follows: complete procedure, repeat procedure, cancel test, finish test. Based on the execution status attributed to procedure, the NPDL will analyze the process algebra expression to decide which procedures can be released to execution. In other words, the software uses the process algebra expression to guide the user through the correct path to perform a genetic test, thereby ensuring that the test was performed according to the desired standards even when different laboratories have defined new tests or test procedures.

Although the NPDL includes all process algebra operators, the CEGH interface was designed to reduce the complexity of genome testing workflows. This is achieved with the implementation of two strategies.

First, as shown in Figure 2, the current CEGH interface maps a set of operators that only are required to perform laboratory genomic testing such as sequential composition “.,” parallel execution “||,” and conditional execution “%.” These operators have proven adequate to support workflow requirements for laboratory genomic testing. If a new genomic test requires the use of additional operators, it is only necessary to adapt the HTML interface. This CEGH interface adaptation is not difficult to implement since all new laboratory genomic test requirements can be represented using the NPDL.

Second, as shown in Figure 3, the current CEGH interface encapsulates mandatory and repetitive tasks, represented by commands such as repetition, cancellation, and termination. This encapsulation is possible as all test procedures must be repeated, cancelled or terminated when a problem occurs or when the results are not conclusive. This HTML adaptation is a disadvantage to the adaptability of the CEGH system. It could be avoided by using a generic or specialized tool for workflow definition. However, the graphical definition of a workflow increases the complexity of workflow definition and requires a trained user with knowledge about workflow notations to model workflows. In summary, with the two strategies of the CEGH interface, end users working at the laboratory do not necessarily have to master the process or scientific workflow notations to design and manage their workflows.

## Conclusions

This paper describes the application of a rigorous approach to event-based PAIS architecture in genetic testing in human genome information systems. Our approach uses a common repository of process planning that ensures reusability, specification, instantiation, monitoring, and execution of processes. Together with a rigorous control flow specification based on the process algebra ACP, an application library implements a workflow engine as SQL extension for process instantiation, execution, control and monitoring which allows taking advantage of relational database scalability and usability of SQL language. It is also presented a real case, the CEGH Information System, to illustrate how our approach is useful to handle scientific processes that are constantly evolving to stay up to date with the latest scientific knowledge. Moreover, the software enables a user to define and control complex processes as he/she understands it without requiring knowledge of business process notation, process algebra definitions or support of computer experts. This system has been used to test more than 100,000 patients and performed 40 different tests to study human Mendelian genetic disorders in the CEGH.

Our ongoing research includes the automatic generation of ACP expressions to genomic complex tests, stochastic process algebra approach to genomic laboratory routines, and handling exceptions to medical and biological information systems.

## Authors' contributions

MRPB and MZ proposed the research hypotheses and described the genetic test components. MRPB tested the software. JEF and LVA designed the software program and the dynamic structure to manage genetic tests. KRB implemented and described the tools for test control. JEF, LVA and SM wrote the manuscript. MRPB, JEF, SM, LVA, and CP reviewed and approved the final manuscript.

## Competing interests

The authors declare that they have no competing interests.
